# HIN2/363: Health Care Resource Guide on the Internet

**DOI:** 10.2196/jmir.1.suppl1.e33

**Published:** 1999-09-19

**Authors:** I Böröcz

**Affiliations:** ^1^Spinerette Information Systems, OrindaUSA

**Keywords:** Medical Informatics, Health Care Resources

## Abstract

**Introduction:**

Presentation of development and creation of a health care resources directory on the Web in the United States and Hungary.

**Methods:**

1. The presentation of health care directory on the Web is hampered by the disparate population in geographic and political subdivisions. A unique concept is developed to collect and present health care resources in sub-units of approximately 1 million people.
2. To permit "needs"-based location of health care resources a list of items is created to present the providers of the desired services.
3. The directory permits linking to the service provider's Web page for easy access of more detailed information.

**Results:**

The system has been completed for a subdivision of California: Diablo Valley. List of institutions, individuals and businesses are listed in logical grouping. The "needs" list includes some 180 items, from "Abortion and Alternative" to "Zen Meditation". Similar lists of hospitals and department head physicians are completed for Hungary. This list will appear also in English and German.

**Discussion:**

The free market requires free choice in health care as well. In order to make choice the alternatives have to be known. The Internet is the perfect approach to present those choices. The project also serves the scientists, practising physicians and other health carer professionals in their ability to communicate with each other.

